# Pericementitis—Its Manifestations and Effects upon the General System

**Published:** 1883-11

**Authors:** G. A. Mills


ARTICLE II.

### PERICEMENTITIS, ITS MANIFESTATIONS IN THE ORAL CAVITY, AND ITS SERIOUS EFFECTS UPON THE GENERAL HEALTH.

(Read, by invitation, before the Kings County Medical Society of Brooklyn, by Dr. G. A. MILLS'S, 169½ Columbia Heights, May 18, 1882.)

Pericementitis, its manifestations in the oral cavity and the serious effects upon the general health, is considered by me of great import to the public whom we are called upon to serve, and certainly should be so considered by us as guardians of the public health. This is the purpose I have in appearing before your body to-night, that I may present to your intelligence facts that will awaken your co-operative interest by taking cognizance of the prevalence of this destructive disorder.

While I fully recognize the fact that the department of the healing art of which it is my pride to be a member is a specialty of the general body, yet I am fully aware of another fact—as you doubtless are—that the attention of the oral cavity, comprising the teeth and their allied structures, has been, in the earlier times, so unnoticed that it created a necessity in the animal economy for the branch or specialty which I represent; and yet while we have the recognition of an unparalleled progress, strange it may seem when I acquaint you with the incontrovertible truth that we have not been able to meet or stay the tide of this cyclone of destructiveness, as yet, to any considerable extent, while the part that has called into action the mechanical ability, has matured into a high degree of excellence. And now the department of education that presides over the culture of surgical ability is being recognized as the important factor and requisite to cope with those of a larger range of cultured ability. So fast as this shall occur in individual or aggregate cases, there will be but one mind regarding the appropriate recognition of fraternal counsel.

The subject which I come to speak to you of is not a new one; it may be, for aught I know, as old as disease itself. It has not been an unnoticed one either, in the general literature of the healing art; it has been characterized under a variety of nomenclature, with which you are more or less familar. So far as my understanding has reached, it has been generally considered as the result of advanced age, and doubtless, for this reason, that the expressions have been the more generally noticed because of the advanced condition so prevalent at this stage of life; while, on the other hand, with the light we now have, it is clearly shown that the mass of cases have their beginnings in early life, and because of the unstrained perception, the minor expressions have failed to gain the notice they now prove to be worthy of. Bleeding and sponginess of the gums and the accumulation of lime, so-called tartar, have been lightly taken into account, and therefore as lightly dealt with, being met simply with some feeble astringent, or by the semi-barbarous operation of scraping or scaling the teeth—of which some of you may still retain a vivid recollection. This being slightly considered, it was found necessary to repeat this inquisition, with frequency, and with little, if any benefit, beyond the comfort of the external surroundings of the teeth and gums; but further on, as a larger identification of complications with personal discomforts associated, the interrogation has been not uncommonly put: "Why this state of affairs? And there no remedy?" And as often received the almost sterotyped answer: "It is not amenable to successful treatment," while untold suffering has accrued, and the loss of thousands upon thousands of teeth that were yet untouched by the disintegrating process of caries. We have often, also, after our best and perfected operations by fillings, been obliged to acknowledge that these efforts were not enough to prove the highest efficiency of our calling; for did it profit anything to be able to save the tooth and, after all, the socket be swept away by disease? Fortunately, out of every great emergency there always seems to be provided some Moses to lead into a larger freedom.

This brings me to a point in this paper which will go into history. I now call your attention to facts that you may not be particularly familar with, and which will serve to answer the question why this subject has come so persistently and hopefully to be considered during the few later years. I hold this particular feature of this paper to be so pertinent and just that I cannot withhold its introduction. To Dr. John M. Riggs, of Hartford, Connecticut, is due the credit of the revival of interest in this subject. A gentleman of no ordinary culture, both as A. B., and M. D., and one who has no peer, in my estimation, in his State, as a true professional man. He has been for some forty years an earnest investigator of the practical details of office practice. His characteristics being somewhat peculiar to himself, he did not come to the arena of discussion in our calling so readily as others, and therefore we did not get the benefit of his observations in this direction so soon as we might otherwise have done. And yet it may be that he came in due time. It may not detract anything of interest to state that Dr. Riggs was the gentleman associated with Dr. Wells, of Hartford, in his experiments with nitrous oxide gas, Dr. Riggs extracting from Dr. Well's mouth a tooth, it being the first operation of this nature under the effect of an anæsthetic ever made. To use the word of the doctor himself, he says: "This disturbance, under consideration, early enlisted his earnest attention," and he came to know that he was meeting with an increasing success beyond that, which he discovered, of his fellow practitioners, and did occasionally drop into limited conversation regarding it, so that it came to be known that he was pursuing a line of treatment not general, if at all known anything of by the profession of dentists.

Dr. Riggs' public announcement of his views created no little credulity and curiosity, they being entirely new and different from anything then incorporated in the general literature. He claims to have found it necessary not only to remove the external deposits about the necks of the teeth· but at a certain stage of the disease to follow on to the margins of the process and trim away that portion that had become a foreign body by inflammatory action, instancing it as a principle recognized in general surgery *i. e.*, to cut back to the life line, or into it, and thus establish a healthy reaction.

I will say, in passing, that human nature is quite the same in our department as in others, and the matter was quietly waived aside by some and vigorously attacked by others—I refer to the older members. Counter claims for originality were put in, but time has not evinced the truth of them ; and I do not hesitate to say that the truth of Dr. Riggs' claim had been fully established to the minds of all fair-minded men who have been cognizant of the discussions that have taken place.

The outcome of this has been the origin of the nomenclature "Riggs' Disease," which has become so familar among us. Dr. Riggs has, as a result of his attention to this subject, devised a set of instruments for treating this disease, so constructed as to be fully able to search out the disturbing points, thus producing results which warrent the saying that it is a surgical operation of no mean order, and one that cannot be familarized without extreme care and intelligent training. A novice can do much harm and afflict his patient severely, while the trained hand, presided over by an intelligent mind, can become an alleviator of great suffering and bring much physical harmony out of decided unhealth.

This subject did not gain the attention of our national body until the session of 1877, held in Chicago. In 1876 and 1877 I published a series of six articles in the *Cosmos* under the title of "What I know about Riggs' Disease." These articles have been very extensively circulated and favorably commented upon in this country and foreign ones. Following these articles Dr. Rehwinkle, of Chillicothe, Ohio, an able writer, presented an article to the national body entitled Pyorrhœa Alveoloris, meaning the pus-discarging sockets, and has also been defined as catarrh of the gums. Before this body the subject was largely and ably discussed by many of our best men, and since that time it has received more or less attention throughout the societies of our specialty, and, as you are aware, occupied a place among the subjects at the International Congress held at London. There it was brought forward under the title of "Premature Wasting of the Alveolar Process." Dr. Riggs was present and engaged in the debate. During the last year the subject was taken up by the Odontological Society of New York City, being introduced by a paper, the product of Dr. Niles, of Boston, a graduate of the Harvard Dental Department, and was christened under the title "The Calcic and Phosphatic Diathesis of Odontolithus." Extensive discussion followed the paper, and both the discussions and the paper may be found in the published proceeding of the society. I have felt that I could not be faithful to this subject without giving some idea of the difficulty and opposition that has encountered a matter of such vast interest and importance, but the cheering thought brings encouragement to those who have labored devoutedly to place this matter in its proper position and to enlarge its sphere of usefulness in the alleviation of human suffering.

[TO BE CONTINUED.]

